# Exploring Nitrogen-Functionalized Graphene Composites for Urinary Catheter Applications

**DOI:** 10.3390/nano13182604

**Published:** 2023-09-21

**Authors:** Rita Teixeira-Santos, Luciana C. Gomes, Rita Vieira, Francisca Sousa-Cardoso, Olívia S. G. P. Soares, Filipe J. Mergulhão

**Affiliations:** 1 LEPABE—Laboratory for Process Engineering, Environment, Biotechnology and Energy, Faculty of Engineering, University of Porto, Rua Dr. Roberto Frias, 4200-465 Porto, Portugal; ritadtsantos@fe.up.pt (R.T.-S.); luciana.gomes@fe.up.pt (L.C.G.); up201603193@edu.fe.up.pt (R.V.); mfcardoso@fe.up.pt (F.S.-C.); 2 ALiCE—Associate Laboratory in Chemical Engineering, Faculty of Engineering, University of Porto, Rua Dr. Roberto Frias, 4200-465 Porto, Portugal; osgps@fe.up.pt; 3 LSRE-LCM—Laboratory of Separation and Reaction Engineering—Laboratory of Catalysis and Materials, Faculty of Engineering, University of Porto, Rua Dr. Roberto Frias, 4200-465 Porto, Portugal

**Keywords:** nitrogen-functionalized graphene, composite, antibiofilm activity, multi-species biofilm, urinary catheters

## Abstract

Graphene has been broadly studied, particularly for the fabrication of biomedical devices, owing to its physicochemical and antimicrobial properties. In this study, the antibiofilm efficacy of graphene nanoplatelet (GNP)-based composites as coatings for urinary catheters (UCs) was investigated. GNPs were functionalized with nitrogen (N-GNP) and incorporated into a polydimethylsiloxane (PDMS) matrix. The resulting materials were characterized, and the N-GNP/PDMS composite was evaluated against single- and multi-species biofilms of *Staphylococcus aureus*, *Pseudomonas aeruginosa*, and *Klebsiella pneumoniae*. Both biofilm cell composition and structure were analyzed. Furthermore, the antibacterial mechanisms of action of N-GNP were explored. The N-GNP/PDMS composite showed increased hydrophobicity and roughness compared to PDMS. In single-species biofilms, this composite significantly reduced the number of *S. aureus*, *P. aeruginosa*, and *K. pneumoniae* cells (by 64, 41, and 29%, respectively), and decreased *S. aureus* biofilm culturability (by 50%). In tri-species biofilms, a 41% reduction in total cells was observed. These results are aligned with the outcomes of the biofilm structure analysis. Moreover, N-GNP caused changes in membrane permeability and triggered reactive oxygen species (ROS) synthesis in *S. aureus*, whereas in Gram-negative bacteria, it only induced changes in cell metabolism. Overall, the N-GNP/PDMS composite inhibited biofilm development, showing the potential of these carbon materials as coatings for UCs.

## 1. Introduction

Indwelling urinary catheters (UCs) are among the most broadly used medical devices for the treatment and mitigation of several clinical conditions. Although UCs are valuable instruments in healthcare settings, they are linked to serious complications, such as urinary tract infections (UTIs). UTIs are the most common type of healthcare-associated infection, with approximately 75% of episodes resulting from the insertion of a UC [1,2]. Catheter-associated urinary tract infections (CAUTIs) have a significant impact on both patient health and the economic burden of healthcare systems [1,3,4].

Polydimethylsiloxane (PDMS), a silicone-based polymer, is widely used for the fabrication of urinary tract devices [5]. Although PDMS presents attractive features, such as mechanical and chemical stability and high biocompatibility, it is particularly susceptible to microbial adhesion [6,7]. In addition, UCs, once inserted, can disrupt the local host defenses and come into contact with various components of the urinary tract (e.g., salts, proteins, and other organic molecules), creating favorable conditions for bacterial attachment and biofilm formation on their surfaces [8,9]. In such intricately organized communities, bacteria are shielded from the host’s immune system and the effects of antimicrobial agents, which helps sustain and facilitate the persistence and dissemination of the infection [10].

CAUTIs can be caused by numerous bacteria, namely *Escherichia coli*, *Staphylococcus aureus*, *Pseudomonas aeruginosa*, *Proteus* spp., *Klebsiella pneumoniae*, and *Enterococcus* spp. [11,12]. In the urinary tract environment, infections often result from multi-species biofilm formation, which usually makes them less susceptible to antibiotic action and, consequently, more challenging to treat [13].

Despite multiple approaches that have been adopted to reduce the risk of UC-related infections, including minimizing unnecessary catheter use, using aseptic techniques during catheter insertion and maintenance, and promptly removing catheters when no longer needed, the incidence of CAUTIs remains high [14]. Coating urinary catheters with metals, such as copper and silver nanoparticles, can provide various benefits, such as reducing infection risks owing to their strong bactericidal action and improving electrical conductivity for therapy [15,16]. During the past few years, a growing interest in the application of carbon materials, such as carbon nanotubes and graphene, as coatings for medical devices has also been reported [17,18].

Graphene consists of a singular layer of carbon atoms organized in a hexagonal lattice arrangement, resulting in a two-dimensional (2D) nanomaterial characterized by remarkable mechanical, electrical, and thermal features [19,20]. Owing to their unique properties, graphene-based materials have been widely applied in biomedicine [21,22,23]. These materials have showcased auspicious antibacterial performance against Gram-negative and Gram-positive bacteria, both in planktonic and biofilm forms [24]. To date, different antimicrobial mechanisms have been proposed for graphene and its derivatives, including the physical piercing of cell membranes and the induction of reactive oxygen species (ROS) production [25,26,27]. However, the antibacterial effectiveness of these carbon materials also depends on the type of bacteria and their morphology [28], the physicochemical properties of the cell surface [29], and their growth state (planktonic or biofilm) [30]. Furthermore, chemical functionalization of graphene with different compounds (e.g., acidic, basic, or natural compounds) has been reported to enhance its antimicrobial properties and biocompatibility [18].

Therefore, considering the importance of controlling biofilm formation on UC surfaces and the antimicrobial potential of graphene materials, the goals of this work were to (1) produce and characterize nitrogen-functionalized graphene nanoplatelets (N-GNP)/polydimethylsiloxane (PDMS) composites, and (2) assess their ability to inhibit single- and multi-species biofilms formed by *S. aureus*, *P. aeruginosa*, and *K. pneumoniae* (three common colonizers of urinary catheters) under conditions prevailing in the urinary tract environment. To gain further insight into the efficacy of the synthesized materials, the antimicrobial mechanisms of action of N-GNP were investigated for the three uropathogenic models. As far as the authors know, this is the first study to apply N-GNP materials in the biomedical field and explore their antimicrobial and antibiofilm potential.

## 2. Materials and Methods

### 2.1. Synthesis of Nitrogen-Functionalized Graphene (N-GNP)

Graphene nanoplatelets (GNP) functionalization with nitrogen (N) groups was performed using melamine as a nitrogen precursor. Pristine GNP (p-GNP; Alfa Aesar, Thermo Fisher Scientific, Erlenbachweg, Germany) (0.6 g) were mixed with 0.39 g of melamine (Sigma-Aldrich Co., Ltd., St. Louis, MO, USA) and ball-milled for 4 h at 15 vibrations s^−1^ using the Retsch MM200 mixer mill equipment (Retsch GmbH, Haan, Germany). The conditions used were based on a previous study [31]. The resulting material (N-GNP) was then exposed to thermal treatment under a N_2_ flow (100 cm^3^ min^−1^) up to 600 °C and maintained at this maximum temperature for 1 h.

### 2.2. Characterization of Pristine and Nitrogen-Functionalized Graphene

#### 2.2.1. Elemental Analysis

The carbon (C), hydrogen (H), nitrogen (N), and sulfur (S) compositions of p-GNP and N-GNP were determined by combustion at 1050 °C using the CHNS mode of Elemental GmbH MICRO equipment. Oxygen (O) analysis was performed via pyrolysis at 1450 °C using OXY equipment (Elemental GmbH, Kalkar, Germany). Each element was calculated as the mean of three independent measurements using a standard compound for calibration [32].

#### 2.2.2. Textural Properties

The textural properties of the graphene materials were based on the N_2_ adsorption isotherms determined using a Quantachrome Nova 4200e apparatus (Quantachrome Instruments, Boynton Beach, FL, USA). The surface areas of p-GNP and N-GNP were estimated by the Brunauer–Emmett–Teller method ($S_{BET}$), and the total pore volume ($V_{p}$) was calculated from the amount of N_2_ uptake at a relative pressure of one. The pore size distribution was assessed using the non-local density functional theory (NLDFT) as described in Gomes et al. [33].

#### 2.2.3. Zeta Potential

The zeta potential of p-GNP and N-GNP suspensions in water was measured using a Malvern Zetasizer Nano (Malvern Instruments Ltd., Malvern, UK) [34].

### 2.3. Production and Characterization of N-GNP/Polydimethylsiloxane (PDMS) Composites

To produce N-GNP/PDMS surfaces, the N-GNP was incorporated at 1 wt. % into SYLGARD™ 184 Silicone Elastomer (Dow Corning, Midland, MI, USA). The selection of N-GNP loading was based on a previous antibiofilm screening assay, which demonstrated that the 1 wt. % N-GNP/PDMS was the most effective composite against 24 h-old biofilms of *S. aureus* compared to 3 and 5 wt. % N-GNP/PDMS surfaces (Figure S1 in Supplementary Materials).

Stirring, sonication, and ultrasonic mixing steps were performed according to the protocol detailed by Oliveira et al. [35]. Then, 30 μL of the N-GNP/PDMS mixture was placed on top of glass (1 × 1 cm; Vidraria Lousada, Lda, Lousada, Portugal) through spin coating (Spin150-v3.2, APT GmbH, Bienenbüttel, Germany), and the coupons were dried overnight in the oven [35], resulting in 1 wt. % N-GNP/PDMS surfaces. Bare PDMS surfaces were produced in a similar fashion and used as a control.

Scanning electron microscopy (SEM), optical profilometry (OP), contact angle measurements, and leaching assays were performed to characterize PDMS and 1 wt. % N-GNP/PDMS surfaces.

#### 2.3.1. SEM

The synthesized surfaces were sputter-coated (ACE600, Leica Microsystems, Wetzlar, Germany) after being mounted on aluminum stubs using carbon adhesive tabs. The surface morphologies were then observed with a secondary electron detector at 3 kV (Zeiss Supra55, Carl Zeiss Microscopy, Oberkochen, Germany).

#### 2.3.2. OP

The surface roughness was analyzed using a non-contact white light profilometer (Proscan 2000, Scantron Industrial Products Ltd., Taunton, UK) as described by Belo et al. [36]. Quadruplicate measurements for each sample were performed (n = 4). Roughness values and representative three-dimensional (3D) plots were obtained using a MATLAB routine (MathWorks, Inc., Natick, MA, USA). The arithmetic mean height of the surface roughness (*S_a_*) represents the mean surface roughness, whereas the root mean square height (*S_q_*) corresponds to the standard deviation of the heights.

#### 2.3.3. Contact Angle Measurements

The contact angles of the synthesized surfaces were measured using the sessile drop technique (Dataphysics OCA 15 Plus, Filderstadt, Germany) with three reference liquids (water, α-bromonaphthalene, and formamide). A minimum of five measurements per liquid and surface were performed in three independent assays (n = 15). The surface free energy (*ΔG*, mJ m^−2^) was then calculated from the contact angles using the van Oss approach [37] as described by Gedas et al. [38] to evaluate the surface hydrophobicity.

#### 2.3.4. Leaching Assays

PDMS (control surface) and 1 wt. % N-GNP/PDMS coupons were placed in 12-well microtiter plates (VWR International, Carnaxide, Portugal), immersed in the Artificial Urine Medium (AUM) [39] used in biofilm formation assays (Section 2.4), and incubated for 24 h at 37 °C without shaking. Then, the content of N-GNP released into solution was determined through ultraviolet–visible (UV–vis) spectral measurements recorded on a JASCO V-560 UV–Vis spectrophotometer (JASCO, Easton, MD, USA).

### 2.4. Biofilm Formation Assay

*S. aureus* (SH1000 expressing GFP), *P. aeruginosa* (PAO1 expressing mCherry), and *K. pneumoniae* (ATCC 13883) were used to assess the antibiofilm and antimicrobial activities of graphene-based surfaces. These three biofilm-forming bacteria are among the most prominent in CAUTIs [12]. Bacteria were stored at −80 °C in Luria-Bertani Broth (LB) medium (Thermo Fisher Scientific, Waltham, MA, USA), and before each experiment, they were spread on Plate Count Agar (PCA; Merck KGaA, Darmstadt, Germany) Petri dishes and incubated at 37 °C. Sterile LB medium, without antibiotic and with chloramphenicol (10 mg L^−1^) or tetracycline (1.25 mg L^−1^), to promote the growth of *K. pneumoniae*, *S. aureus*, and *P. aeruginosa*, respectively, was then inoculated with individual colonies from the PCA plates and incubated overnight at 37 °C in a shaker at 160 rpm (Grant Bio™ PSU-10i, Fisher Scientific, Leicestershire, UK). After centrifugation at 2576× *g* for 10 min (Eppendorf Centrifuge 5810R, Eppendorf, Hamburg, Germany), pellets were resuspended in fresh AUM to obtain bacterial suspensions with an optical density of 0.1 at 610 nm (approximately 1 × 10^7^ cells mL^−1^).

Biofilm formation assays were performed using 12-well plates and duplicate coupons of 1 wt. % N-GNP/PDMS and PDMS (control surface). First, the microtiter plates and the coupons glued to the bottom of the wells were sterilized by UV light [38]. To form single-species biofilms, 3 mL of each suspension (*S. aureus*, *P. aeruginosa*, or *K. pneumoniae*) was placed into the microplate wells. For multi-species biofilms, the three bacterial inocula were added at a 1:1:1 ratio, having a final density of 1 × 10^7^ cells mL^−1^. Additionally, 3 mL of sterile AUM was added to a microwell to control sterility throughout the assay (negative control). The plates were then incubated under static conditions for 24 h at 37 °C. Six independent assays were carried out, each with two technical replicates (n = 12).

### 2.5. Biofilm Analysis

In single- and multi-species biofilms, the quantity of total and culturable cells was gauged using flow cytometry and plate counts, respectively. Moreover, the biofilm arrangement and population composition were studied through confocal laser scanning microscopy (CLSM).

#### 2.5.1. Total Cell Count

The coupons were removed from the microplate wells, submerged in 2 mL of 8.5 g L^−1^ NaCl sterile solution, and vortexed for 2 min at maximum speed to obtain biofilm cell suspensions. They were analyzed in a CytoFLEX flow cytometer model V0-B3-R1 (Beckman Coulter, Brea, CA, USA) with the CytExpert software (version 2.4.0.28, Beckman Coulter, Brea, CA, USA). Bacteria were gated based on their side scatter (SSC) and forward scatter (FSC) signals. For single-species biofilms, cells were identified based on their size and complexity in an FSC versus SSC plot. For multi-species biofilms, the three pathogens were identified based on the fluorescent events registered at FL1 (fluorescence detector; bandpass (BP) filter 525/40 nm), which correspond to *S. aureus* expressing GFP; on the fluorescent events recorded at FL2 (BP 585/42 nm), which correspond to *P. aeruginosa* expressing mCherry; and on non-fluorescent events, which correspond to *K. pneumoniae*, along with the pathogens’ size and complexity features (SSC versus FSC plot). Subsequently, 10 µL of bacterial suspension was acquired. Sample acquisition was conducted at a flow rate of 10 µL min^−1^. The results are presented as cells per cm^2^.

#### 2.5.2. Culturable Cell Count

The number of culturable cells per cm^2^ was determined by colony-forming unit (CFU) count. Serial dilutions of biofilm suspensions were performed and spread on PCA (for *K. pneumoniae* enumeration) and PCA supplemented with tetracycline (for *P. aeruginosa* colony selection) and chloramphenicol (for *S. aureus* colony selection). The plates were then incubated at 37 °C for 24 h.

#### 2.5.3. Confocal Laser Scanning Microscopy (CLSM) and Image Analysis

Biofilm cells were stained with 0.5 mg⋅L^−1^ of 4′,6-diamidino-2-phenylindole (DAPI; Invitrogen Life Technologies, Alfagene, Portugal), a blue fluorescent DNA stain, for 10 min in the dark. Biofilm samples were then inverted, mounted on a coverslip, and analyzed using a Leica DMI6000-CS inverted microscope (Leica Microsystems, Wetzlar, Germany) with a ×40 water immersion objective (Leica HCX PL APO CS; Leica Microsystems). GFP, mCherry, and DAPI signals were simultaneously collected using differential excitation and emission wavelengths. To visualize GFP expressed by *S. aureus* biofilm cells, a 488 nm argon laser was used for excitation in combination with a 500–550 nm bandpass emission filter. The mCherry protein produced by *P. aeruginosa* was detected using a 633 nm helium-neon laser in combination with a 610–680 nm bandpass emission filter. DAPI-stained cells were observed with a 405 nm diode-UV laser line and a 350–470 nm bandpass emission filter. A minimum of five stacks of horizontal plane images (387.5 μm × 387.5 μm) with a *z*-step of 1 μm was acquired for each biofilm sample. In the confocal images presented, *S. aureus*, *P. aeruginosa*, and *K. pneumoniae* cells are shown in green, red, and blue, respectively.

Simulated 3D projections and two-dimensional (2D) sections of the biofilms were generated using IMARIS 9.3 (Bitplane AG, Zurich, Switzerland). Biofilm parameters, namely biovolume (µm^3^ µm^−2^) and thickness (µm), were extracted from CLSM stacks using COMSTAT2 [40]. In multi-species biofilms, the relative abundance of each bacterial strain was determined from the biovolume fractions.

### 2.6. Evaluation of N-GNP Mechanisms of Action

The mechanisms of N-GNP against *S. aureus*, *P. aeruginosa*, and *K. pneumoniae* were characterized using flow cytometry. Bacterial suspensions (1 × 10^7^ cells mL^−1^) were exposed to 1% (*w*/*v*) N-GNP for 24 h at 37 °C; non-treated cells were used as controls. Cells were harvested by centrifugation and the supernatant was collected for analysis [38].

Cell membrane potential, cell membrane integrity, bacterial metabolic activity, and ROS production were evaluated by staining the cells with bis-(1,3-dibutylbarbituric acid) trimethine oxonol (DiBAC_4_(3); Sigma-Aldrich, Taufkirchen, Germany) at 0.5 µg mL^−1^ [41], propidium iodide (PI, Invitrogen Life Technologies, Alfagene, Lisboa, Portugal) at 1 μg mL^−1^ [42], 5(6)-carboxyfluorescein diacetate (5-CFDA; Sigma-Aldrich, Taufkirchen, Germany) at 5 µg mL^−1^ [42], and 2′,7′-dichlorofluorescein diacetate (DCFH-DA, Sigma-Aldrich, Taufkirchen, Germany) at 10 µM [43], respectively.

Bacterial suspensions were stained for 30 min in the dark and 10,000 events were analyzed at a flow rate of 10 µL min^−1^. The results are presented as the mean intensity of fluorescence (MIF) at FL1 for DiBAC_4_(3), 5-CFDA and DCFH-DA, and at FL2 for PI.

### 2.7. Statistical Analysis

Data were analyzed with IBM SPSS Statistics version 29.0 for Microsoft (IBM SPSS, Inc., Chicago, IL, USA). Descriptive statistics were employed to compute the mean and standard deviation (SD) for elemental analysis, surface roughness parameters, contact angles, number of biofilm cells, and biofilm biovolume and thickness. Differences in biofilm cell numbers according to the type of surface were evaluated through Kruskal–Wallis and Mann-Whitney tests, given the non-normal distribution of the variables. In turn, quantitative parameters obtained from the CLSM analysis (biofilm biovolume and thickness) were compared using one-way analysis of variance (ANOVA). Statistically significant differences were considered for *p*-values < 0.05.

## 3. Results and Discussion

### 3.1. Characterization of Pristine and Nitrogen-Functionalized Graphene

To understand whether graphene surface modification with nitrogen was successfully achieved, elemental analysis of N-GNP was performed and compared to p-GNP (Table 1).

Data revealed that GNP consisted mostly of carbon (90.8%), followed by oxygen and hydrogen (5.6 and 0.4%, respectively), which is supported by the literature [32]. Similarly, N-GNP was mostly composed of carbon (88.6%) and presented low amounts of oxygen (3.8%) and hydrogen (0.6%). In contrast to p-GNP, it was possible to detect considerable amounts of nitrogen (4.2%) in N-GNP. This result indicated that the treatment with melamine through a solventless method using ball milling introduced nitrogen on the surface of GNP. Previous studies have reported that treatment with N-precursors results in the incorporation of a considerable amount of nitrogen (between 0.2% and 4.8%) on the graphene surface [32], and that the N-functionalities include mainly pyridinic-N (N-6), pyrrolic-N (N-5), and quaternary nitrogen (N-Q) in lower amounts [31,32]. In addition, by incorporating nitrogen groups into GNP structures, it is expected that some carbon atoms can be replaced [32]. Altogether, these results confirm that N incorporation on the GNP surface was achieved.

**Figure 1:**
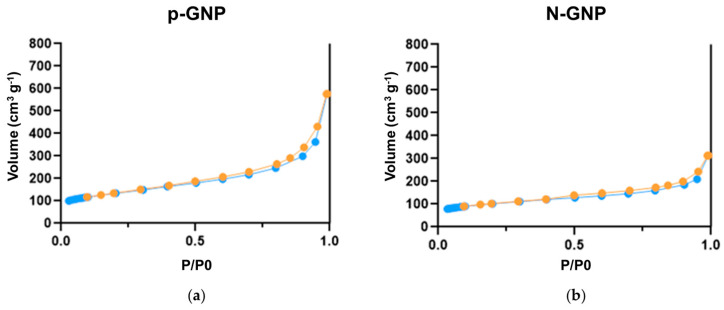
Nitrogen adsorption–desorption isotherms of (**a**) p-GNP and (**b**) N-GNP determined at −196 °C. The orange dots (●) represent adsorption, while the blue dots (●) represent desorption from the particles.

The N_2_ adsorption–desorption isotherms for the p-GNP and N-GNP materials are shown in Figure 1. According to IUPAC, they can be classified as type II [44,45], which is characteristic of carbon materials with some mesoporosity [33].

Through adsorption–desorption isotherm analysis at −196 °C, it was possible to determine the textural properties of each sample. The specific surface area (S_BET_), the volume of micropores (V_micro_), the surface area of mesopores (S_meso_), and the total pore volume (V_p_) were determined (Table 2).

For the N-GNP sample, the amount of N_2_ adsorbed was lower than that for the p-GNP sample (Figure 1). This may be a consequence of the different degrees of agglomeration of the graphene sheets in the two materials [31].

In turn, there was a decrease in the surface area (S_BET_) of p-GNP to N-GNP, from 471 to 361 m^2^ g^−1^. This is probably because the N-GNP underwent a mechanical treatment and was functionalized with a nitrogen source, decreasing its surface area when exfoliating and cutting the graphene sheets [46].

Regarding the surface area of the mesopores (S_meso_), N-GNP presented a lower value compared to p-GNP (196 versus 353). In contrast, N-GNP V_micro_ slightly increased compared to p-GNPs (0.073 versus 0.052). This may be because graphene sheets do not have as much space between them due to nitrogen incorporation [31].

Altogether, these results suggest that ball milling and heat treatments (600 °C) applied to the GNP material during N functionalization promoted several modifications in their textural and chemical properties, mainly due to the exfoliation of graphene sheets [46]. In addition, it can be concluded that the N-doping of GNP with melamine results in a decreased surface area compared to the p-GNP material, which suggests that the addition of the N-precursor contributes to greater aggregation of the graphene sheets.

The surface charges of p-GNP and N-GNP were also evaluated by measuring the zeta potential. N-GNP displayed a zeta potential of −4.93 ± 0.45 mV, while p-GNP of −6.42 ± 0.34 mV. Thus, both graphene samples had a slightly negative charge and may experience electrostatic repulsion when interacting with negatively charged bacterial cell surfaces. This repulsion can hinder direct contact between GNP and bacterial cells, making their interaction less favorable.

### 3.2. Composite Characterization

#### 3.2.1. Surface Morphology

**Figure 2:**
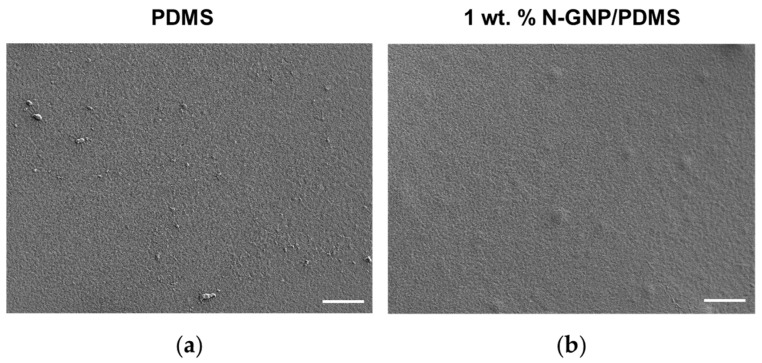
SEM micrographs of (**a**) PDMS and (**b**) 1 wt. % N-GNP/PDMS composites. The images have a magnification of 500× and the scale bars are 50 µm.

It is well known that the morphology of nanocomposite films has an impact on the extent of bacterial cell adhesion [47,48]. Thus, the surface morphology of PDMS (control) and 1 wt. % N-GNP/PDMS was visualized by electron microscopy (Figure 2). While PDMS displayed a homogeneous surface (Figure 2a), the 1 wt. % N-GNP/PDMS composite exhibited protrusions scattered across the surface area under analysis (Figure 2b). These irregularities correspond to graphene agglomerates that form small elevations on the coating surface, as previously observed by the group for pristine GNP [35,38]. The presence of graphene clusters on the surfaces could enhance their effectiveness as they encourage direct interaction between the carbon nanomaterial and bacterial cells, which, in turn, can amplify the antibacterial efficacy of these composites [27,49].

#### 3.2.2. Surface Roughness

**Figure 3:**
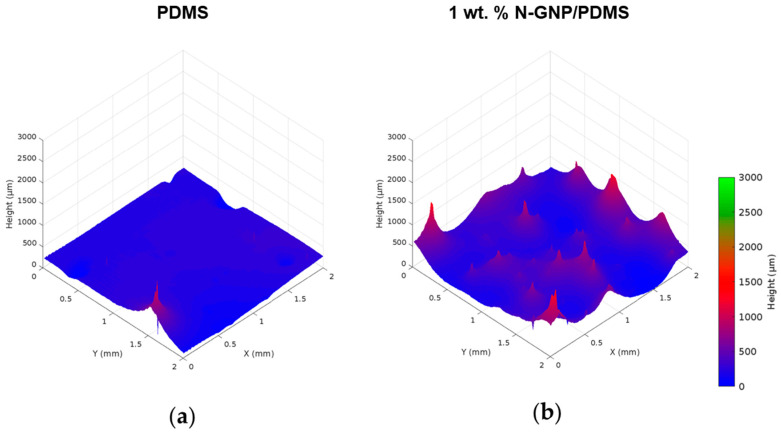
Profilometry plots of (**a**) PDMS and (**b**) 1 wt. % N-GNP/PDMS surfaces.

The roughness of both surfaces was determined by optical profilometry. Looking at Figure 3, it is possible to observe an increase in roughness from PDMS (Figure 3a) to 1 wt. % N-GNP/PDMS (Figure 3b). Quantitative data (Table 3) confirmed this by revealing that the incorporation of N-GNP into the PDMS matrix increased the *S_a_* and *S_q_* values by a factor of approximately 1.5. These results corroborated the SEM analysis, in which PDMS showed the smoothest surface (Figure 2), and suggest that graphene-based surfaces may promote biofilm growth since surface roughness increases the area available for bacterial attachment [47].

#### 3.2.3. Surface Hydrophobicity

Hydrophobicity can also influence the attachment of bacteria by providing suitable substrata for their adhesion or by hindering their ability to attach [47]. Contact angle measurements provide a theoretical prediction of microbial adhesion on the tested surfaces by determining the degree of hydrophobicity. Analyzing the results in Table 4, both tested surfaces are hydrophobic, as they presented water contact angles higher than 90°, as well as negative values of free energy of interaction (*ΔG*). In turn, the 1 wt. % N-GNP/PDMS surface showed higher hydrophobicity than PDMS (i.e., lower *ΔG* value), meaning that the interactions between the N-GNP nanosheets were stronger than those with water.

According to several studies, hydrophobic surfaces are more susceptible to bacterial adhesion [50,51]. However, other studies have not found a direct link between surface hydrophobicity and bacterial cell adhesion [52]. These contradictory results can be explained by other aspects, including biological (e.g., the presence of cell structures such as flagella) and environmental factors, which alter the adhesion conditions of microorganisms [53]. Furthermore, biofilm development on the surface can trigger alterations in the hydrophobic properties of the substrate. Hence, these findings alone do not provide sufficient evidence to accurately assess the extent of biofilm formation by the tested bacteria on the fabricated surfaces.

### 3.3. Antibiofilm Performance of N-GNP/Polydimethylsiloxane (PDMS) Composites

To unveil the antibiofilm and antimicrobial activities of the N-GNP/PDMS composite, uropathogenic biofilms were formed at 37 °C for 24 h under static conditions. These conditions mimic those of the colonization of the extraluminal side of UCs and the balloon in the case of a Foley catheter, which are exposed to quasi-static urine in the bladder [54]. Biofilms were analyzed in terms of the number of total and culturable cells as well as their architecture (biovolume and thickness).

#### 3.3.1. Biofilm Total Cells

**Figure 4:**
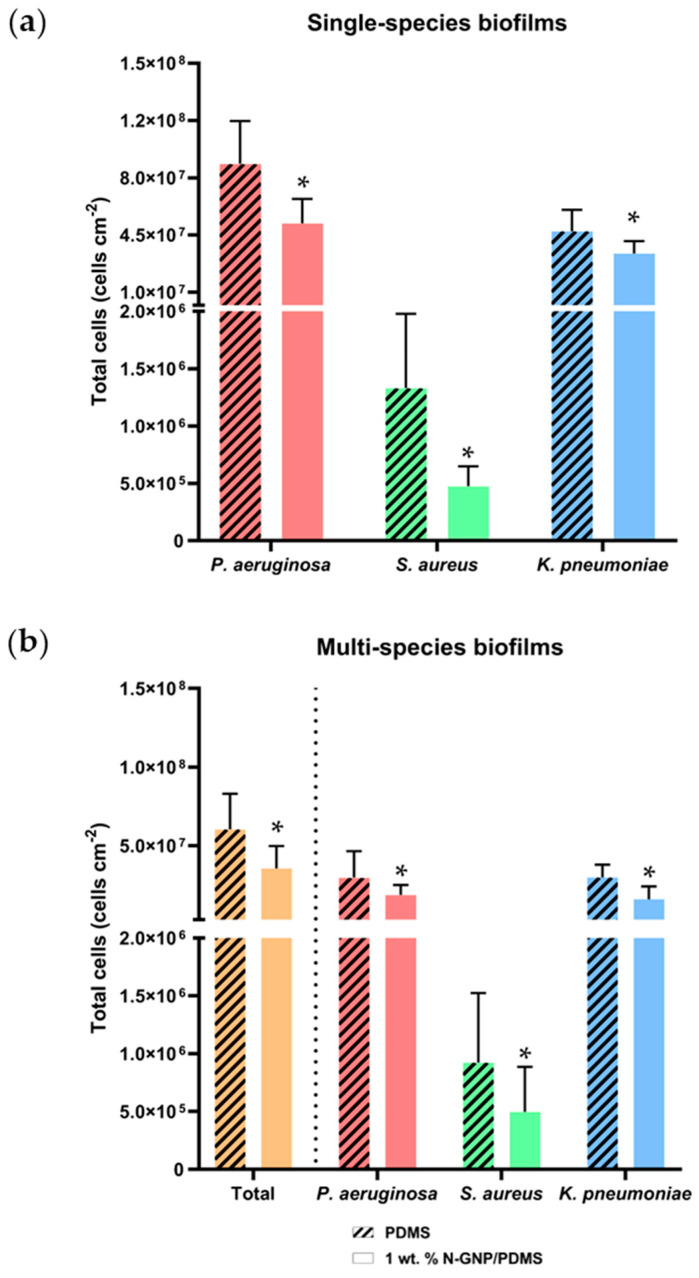
Number of total cells of (**a**) single- and (**b**) multi-species biofilms formed by *P. aeruginosa*, *S. aureus*, and *K. pneumoniae* on PDMS (striped bars) and 1 wt. % N-GNP/PDMS (clear bars) surfaces. Results are presented as mean ± SD. Differences between surfaces were considered significant at *p* < 0.05 (*).

The total biofilm cell number determined by flow cytometry confirmed that the uropathogenic bacteria tested were able to adhere to and form biofilms on the synthesized surfaces under the tested conditions (Figure 4). The results indicated that *P. aeruginosa* was the strongest biofilm-forming bacterium, whereas *S. aureus* was the weakest (*p* < 0.001).

For single-species biofilms (Figure 4a), both *P. aeruginosa* and *K. pneumoniae* biofilms formed on 1 wt. % N-GNP/PDMS surfaces showed a significantly lower number of total biofilm cells than PDMS (5.2 × 10^7^ versus 8.9 × 10^7^ cell cm^−2^ and 3.4 × 10^7^ versus 4.7 × 10^7^ cell cm^−2^ for *P. aeruginosa* and *K. pneumoniae*, respectively). Interestingly, 1 wt. % N-GNP/PDMS surfaces strongly reduced the number of *S. aureus* biofilm cells when compared to the control surface (4.8 × 10^5^ versus 1.3 × 10^6^ cell cm^−2^; *p* < 0.001). Overall, total cell reductions of 41, 64, and 29% were obtained for *P. aeruginosa*, *S. aureus*, and *K. pneumoniae*, respectively, on the N-GNP/PDMS surface. For multi-species biofilms (Figure 4b), the 1 wt. % N-GNP/PDMS surface showed a significantly lower number of total biofilm cells than PDMS (41% reduction). Furthermore, the numbers of *P. aeruginosa*, *S. aureus*, and *K. pneumoniae* adhered to 1 wt. % N-GNP/PDMS surfaces were significantly lower compared to PDMS (1.9 × 10^7^ versus 3.0 × 10^7^ cells cm^−2^ for *P. aeruginosa*, 5.0 × 10^5^ versus 9.2 × 10^5^ cells cm^−2^ for *S. aureus*, and 1.6 × 10^7^ versus 3.0 × 10^7^ cells cm^−2^ for *K. pneumoniae*). In general, biofilm cells of *P. aeruginosa* were reduced by 37%, and biofilm cells of *S. aureus* and *K. pneumoniae* were reduced by 46% on a 1 wt. % N-GNP/PDMS surface. These results demonstrate the antibiofilm performance of nitrogen-functionalized GNP surfaces against single- and multi-species biofilms. Although surface characterization analysis suggested that 1 wt. % N-GNP/PDMS could be more susceptible to bacterial adhesion and biofilm formation, factors other than surface properties, such as bacterial surface characteristics, probably contributed to its antibiofilm performance.

Considering the bacterial population in multi-species biofilms formed on the N-GNP/PDMS surface, *P. aeruginosa* was the predominant bacteria, followed by *K. pneumoniae* and *S. aureus* (Figure S2 in Supplementary Materials). This may explain the similarity in cell reduction between single-species biofilms of *P. aeruginosa* and multi-species biofilms (both around 41%).

#### 3.3.2. Biofilm Culturable Cells

**Figure 5:**
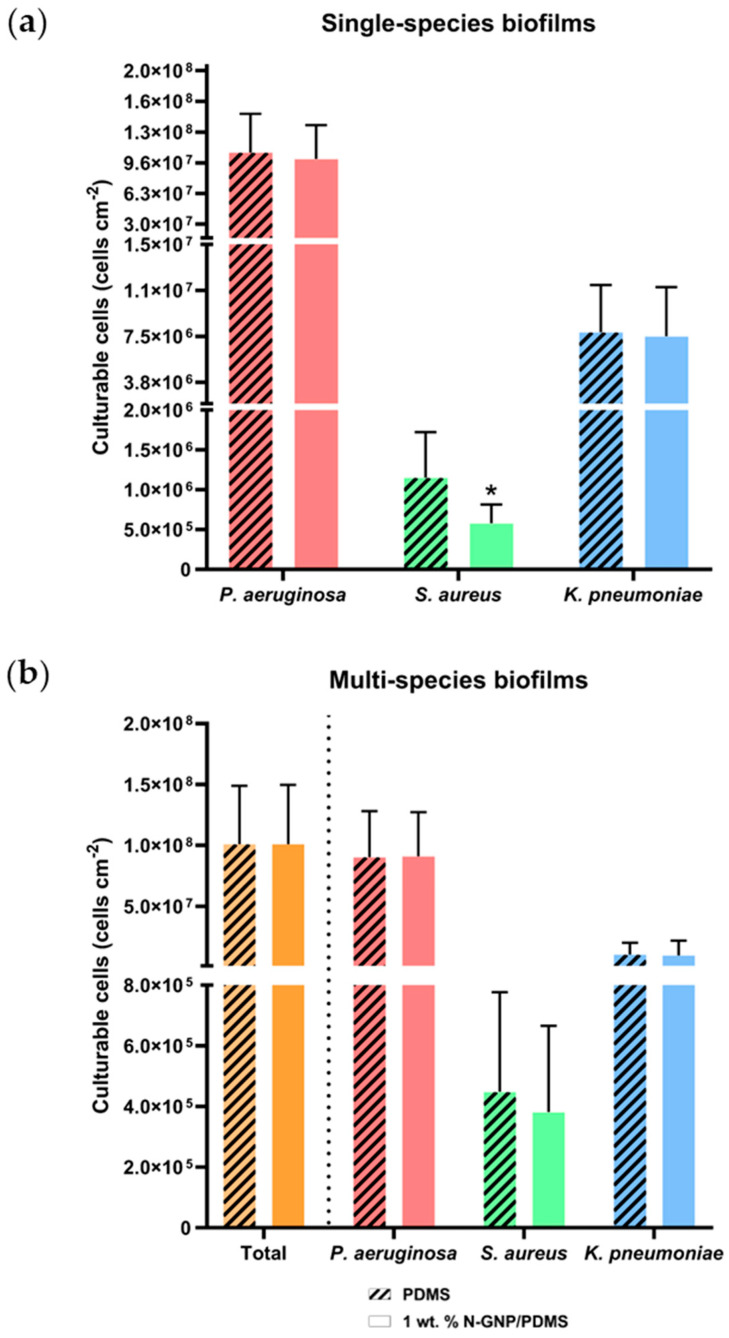
Number of culturable cells of (**a**) single- and (**b**) multi-species biofilms formed by *P. aeruginosa*, *S. aureus*, and *K. pneumoniae* on PDMS (striped bars) and 1 wt. % N-GNP/PDMS (clear bars) surfaces. Results are presented as mean ± SD. Differences between surfaces were considered significant at *p* < 0.05 (*).

Figure 5 shows the number of culturable cells per unit surface area determined by CFU counting for single- and multi-species biofilms. Antibiotic-selective agar plates were used to distinguish between the three bacterial strains chosen for the growth of mixed biofilms.

Concerning single-species biofilms (Figure 5a), the N-GNP/PDMS surface showed a significantly lower number of *S. aureus* cells than PDMS (5.8 × 10^5^ versus 1.2 × 10^6^ cells cm^−2^; *p* = 0.007). However, for *P. aeruginosa* and *K. pneumoniae* biofilms, the graphene-based surface had a similar number of culturable cells to control (*p* > 0.05). Culturable cell reductions of 7, 50, and 4% were obtained for *P. aeruginosa*, *S. aureus*, and *K. pneumoniae*, respectively, indicating a high antimicrobial performance of the nitrogen-functionalized GNP composites against Gram-positive cells and a lack of activity against Gram-negative bacteria.

Regarding culturable cells in multi-species biofilms (Figure 5b), the results followed the same trend. The 1 wt. % N-GNP/PDMS surfaces showed an equal number of sessile cells to that of PDMS (around 1.0 × 10^8^ cells cm^−2^). Moreover, there were no significant differences between the number of culturable *P. aeruginosa*, *S. aureus,* and *K. pneumoniae* cells adhered to N-GNP/PDMS and PDMS surfaces. Comparing the performance of the N-GNP/PDMS surface with PDMS, only a 15% culturable cell reduction was obtained for *S. aureus* in multi-species biofilms. Altogether, these results indicate that the N-GNP/PDMS composite only exhibited antimicrobial activity against *S. aureus*, and this activity decreased when multi-species biofilms were considered.

These findings align with prior research that showed a more pronounced antibacterial effect of graphene on Gram-positive bacteria compared to Gram-negative bacteria [24,55,56,57]. Gram-positive bacteria, such as *S. aureus*, feature a cytoplasmic membrane enveloped by a peptidoglycan layer. Conversely, Gram-negative bacteria such as *P. aeruginosa* and *K. pneumoniae* possess a more intricate cell envelope encompassing a plasma membrane, a peptidoglycan cell wall, and an outer membrane primarily composed of lipopolysaccharide (LPS) [58]. It has been hypothesized that this outer membrane largely contributes to the rigidity and resistance of Gram-negative cells [59,60]. This could potentially hinder cell membrane rupture, which is considered one of the primary mechanisms of action of graphene materials.

Concerning bacterial interactions, as the number of culturable cells for each tested species was similar to that obtained for single-species biofilms, a neutral relationship between the different strains likely occurred (Figure 5).

#### 3.3.3. CLSM Analysis

The antibiofilm performance of N-GNP composites against single- and tri-species biofilms of *P. aeruginosa*, *S. aureus*, and *K. pneumoniae* was evaluated using confocal microscopy.

**Figure 6:**
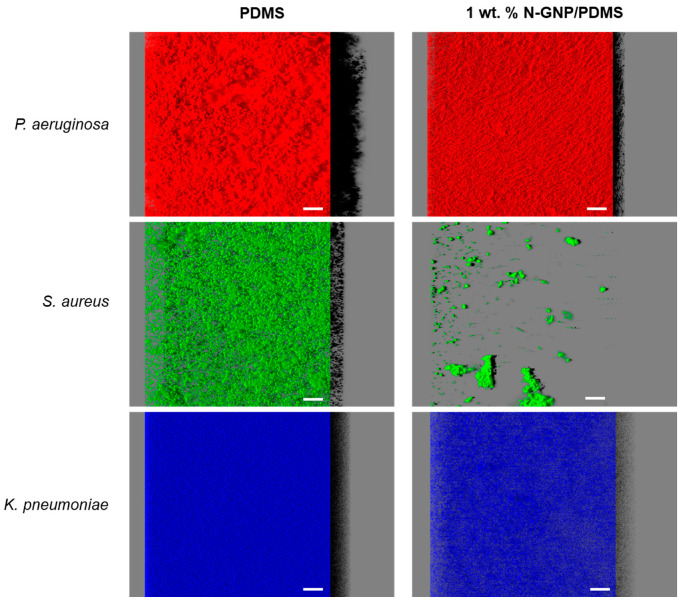
Single-species biofilms of *P. aeruginosa* (**top row**), *S. aureus* (**middle row**), and *K. pneumoniae* (**bottom row**) developed on PDMS (**left column**) and 1 wt. % N-GNP/PDMS (**right column**) surfaces. These representative images were obtained using the IMARIS 9.3 software and present an aerial, 3D view of the biofilms. The black shadow on the right represents the vertical projection of biofilm. The white scale bars are 40 μm.

Figure 6 illustrates the three-dimensional (3D) arrangement of single-species biofilms of *P. aeruginosa*, *S. aureus*, and *K. pneumoniae* formed on PDMS (control surface) and PDMS incorporated with nitrogen-functionalized graphene nanoplatelets (N-GNP/PDMS). It was observed that *P. aeruginosa* formed denser and thicker biofilms (shadow projections on the right of the images; top row of Figure 6) than *S. aureus* and *K. pneumoniae*, and this was particularly noticeable in biofilms developed on PDMS. Here, the resulting *P. aeruginosa* biofilm had a heterogeneous architecture, covering the entire surface with mounds of various heights. Among the two bacterial strains that formed less biofilm (*S. aureus* and *K. pneumoniae*), *K. pneumoniae* (bottom row of Figure 6) formed 24 h biofilms with greater surface coverage and thickness than *S. aureus* (middle row of Figure 6), despite the tested surface material.

Regarding the surface effect, 1 wt. % N-GNP/PDMS (Figure 6, right column) showed the lowest biofilm amount compared to PDMS (Figure 6, left column), regardless of the bacterial strain tested. This reduction in biofilm mass between PDMS and functionalized PDMS was particularly evident for the Gram-positive strain. Indeed, while a loosely packed, open biofilm architecture of *S. aureus* was detected on PDMS, a few heterogeneously distributed clusters of cells were observed on the N-GNP surface, sharply decreasing the surface area covered by biomass.

**Figure 7:**
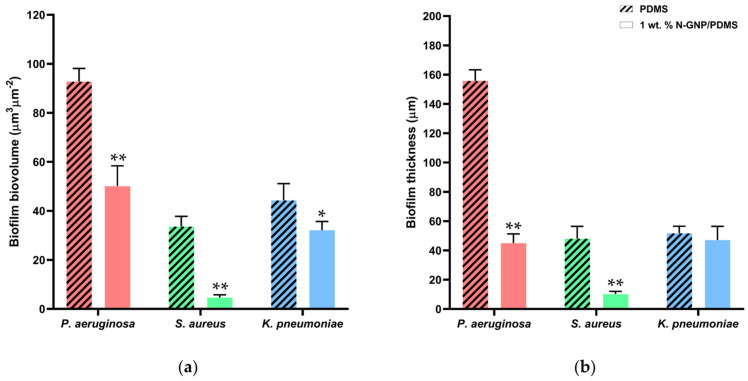
(**a**) Biovolume and (**b**) thickness of single-species biofilms of *P. aeruginosa*, *S. aureus*, and *K. pneumoniae* formed on PDMS (striped bars) and 1 wt. % N-GNP/PDMS (clear bars) surfaces obtained from confocal image series. The means ± SDs for three independent experiments are presented. Significant differences were considered for *p* < 0.05 by (*) and < 0.001 by (**).

Biofilm biovolume and thickness values appraised from confocal image analysis validated these qualitative findings (Figure 7). The N-GNP surface reduced the biovolume of *P. aeruginosa*, *S. aureus*, and *K. pneumoniae* single-species biofilms by 46, 86, and 28%, respectively, compared to the PDMS surface (*p* < 0.05, Figure 7a). Additionally, there was a reduction of approximately 75% in the biofilm thickness of *P. aeruginosa* and *S. aureus* between both surfaces (*p* < 0.001, Figure 7b), whereas the thickness of *K. pneumoniae* biofilms decreased by only 9% on the N-GNP compared to the control (*p* > 0.05, Figure 7b). These results confirmed the greater potential of N-GNP composites for the prevention of staphylococcal biofilms in the urinary context, which was previously demonstrated by flow cytometry data (Figure 4).

**Figure 8:**
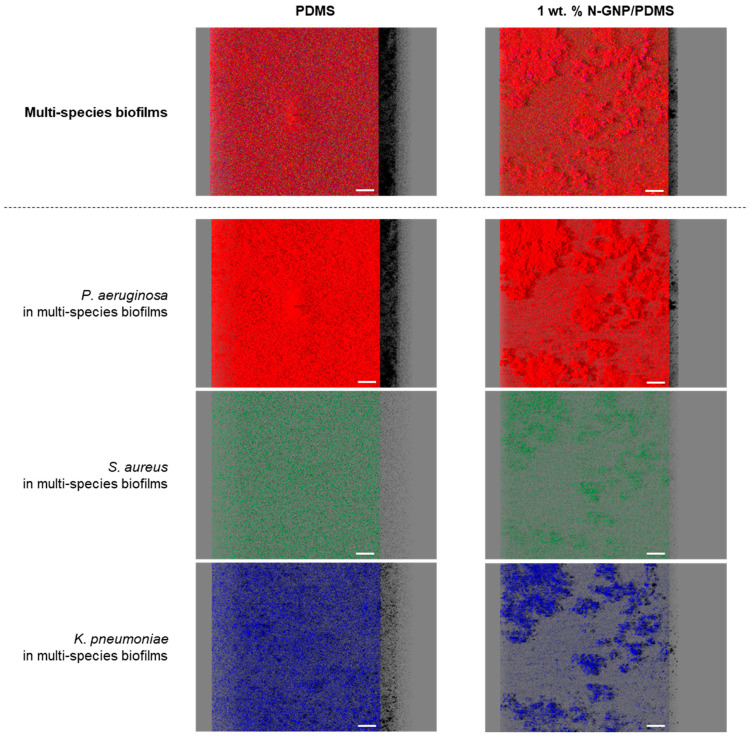
Multi-species biofilms of *P. aeruginosa*, *S. aureus*, and *K. pneumoniae* formed on PDMS (**left column**) and 1 wt. % N-GNP/PDMS (**right column**) surfaces. *P. aeruginosa* was labeled in red, *S. aureus* was labeled in green, and *K. pneumoniae* was counterstained in blue with DAPI. The first row corresponds to the overlap of red, green, and blue channels (*P. aeruginosa* + *S. aureus + K. pneumoniae*), while the other rows are the individual channel compositions. Each image is an aerial, 3D view of the biofilms obtained as in Figure 6. The white scale bars represent 40 μm.

**Figure 9:**
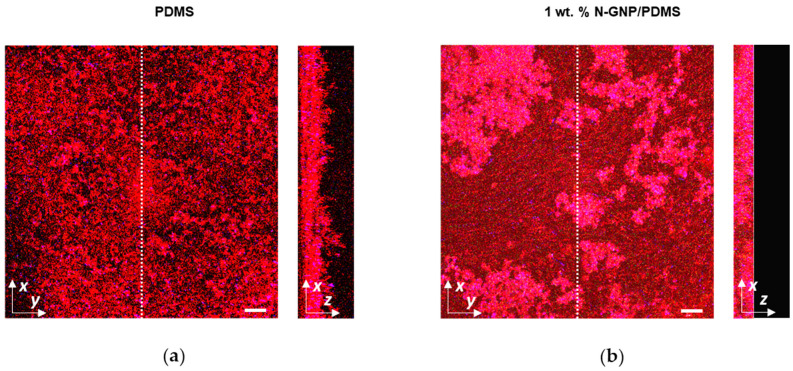
Bacterial distribution in multi-species biofilms formed on (**a**) PDMS and (**b**) 1 wt. % N-GNP/PDMS surfaces. *P. aeruginosa* was labeled in red, *S. aureus* was labeled in green, and *K. pneumoniae* was counterstained in blue with DAPI. These are top and vertical 2D sectional views of the CLSM images presented in Figure 8. The top micrographs correspond to the top view of the biofilm, while the micrographs shown on the right side are vertical sections of the biofilm collected at the positions indicated by the white dots. Purple regions appear because the red and blue colors superimpose. The white scale bars represent 100 μm.

Three- and two-dimensional representations of tri-species biofilms of *P. aeruginosa*, *S. aureus*, and *K. pneumoniae* grown on PDMS and N-GNP/PDMS are displayed in Figure 8 and Figure 9, respectively. This microscopic study was crucial for elucidating the interactive behavior of the strains when cultured for 24 h. Regardless of the surface material, consortium biofilms were mainly composed of *P. aeruginosa* cells (second row of Figure 8 and Figure 9). The second bacterial strain that appears to be present in greater numbers in both biofilms is *K. pneumoniae* (last row of Figure 8), as several purple regions that are due to overlapping red and blue fluorescent signals were visible in 2D confocal images (Figure 9). In turn, residual amounts of *S. aureus* cells (marked in green) were randomly detected within the tri-species biofilms (third row of Figure 8 and Figure 9). These results are supported by the analysis of the biofilm total and culturable cells (Figure 4 and Figure 5, respectively). With regard to the differences between surfaces, in general, the biofilm formed on PDMS had a higher cell density and thickness than that developed on PDMS with N-GNP, similar to what was observed for single-species biofilms (Figure 6). Moreover, the biofilms formed on PDMS exhibited structures characterized by a more consistent thickness compared to those developed on the N-GNP/PDMS surface, where several cell agglomerates stand out (first row of Figure 8 and Figure 9). The CLSM study also indicated that there was no noticeable preference for spatial localization of the three bacterial strains along the biofilm thickness (Figure 9).

**Figure 10:**
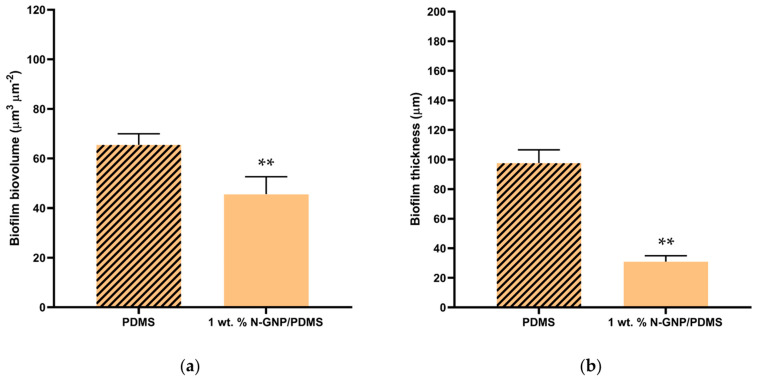
(**a**) Biovolume and (**b**) thickness of tri-species biofilms formed on PDMS and 1 wt. % N-GNP/PDMS obtained from confocal image series. The means ± SDs for three independent experiments are presented. Significant differences were considered for *p* < 0.001 (**).

The average values extracted for the total biovolume and thickness of these multi-species biofilms (Figure 10) reinforced the visual observation (Figure 8 and Figure 9). There were significant reductions of approximately 30% and 70% in biofilm biovolume and thickness, respectively, in the tri-species biofilms formed on modified PDMS compared to PDMS (*p* < 0.001).

**Figure 11:**
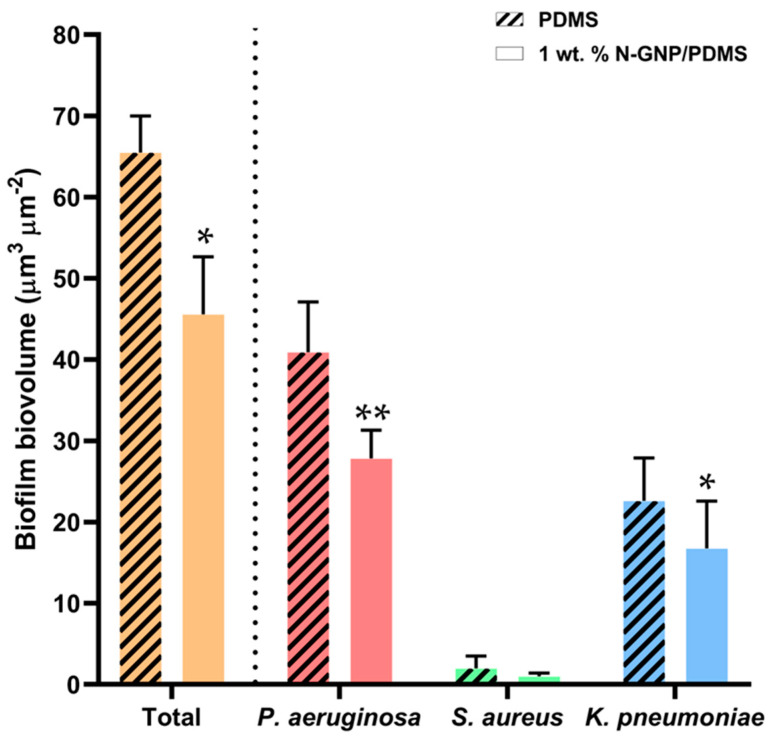
Total biovolume and biovolume of *P. aeruginosa*, *S. aureus*, and *K. pneumoniae* in tri-species biofilms formed on PDMS (striped bars) and 1 wt. % N-GNP/PDMS (clear bars) surfaces obtained from confocal image series. The means ± SDs for three independent experiments are presented. Significant differences were considered for *p* < 0.05 by (*) and < 0.001 by (**).

Regarding the presence of each bacterial strain within the tri-species biofilms, the quantitative data (Figure 11) confirmed that *P. aeruginosa* was the dominant strain on both surfaces (making up, on average, 62% of the total biovolume), followed by *K. pneumoniae* (up to 35% of the total biovolume), and finally *S. aureus* (only 3% of the total biovolume). Looking at the influence of the surface material on the species proportion, the decrease in *P. aeruginosa* and *K. pneumoniae* biomass in the functionalized graphene composite was on average 29% compared to bare PDMS (*p* < 0.05), which is in line with the reduction percentage of the total biovolume. However, the surface seemed to have had a greater effect on the *S. aureus* population within the multi-species biofilms (49% reduction on N-GNP/PDMS compared to PDMS).

Overall, the antibiofilm effectiveness of N-GNP-based surfaces was confirmed through both the analysis of biofilm cell composition and the assessment of biofilm structure.

The short-term stability of the composites was also evaluated under in vitro conditions, and no leaching of the functionalized graphene was detected (Figure S3 in Supplementary Materials), reinforcing the potential of this type of coating for urinary applications.

### 3.4. N-GNP Mechanisms of Action

**Figure 12:**
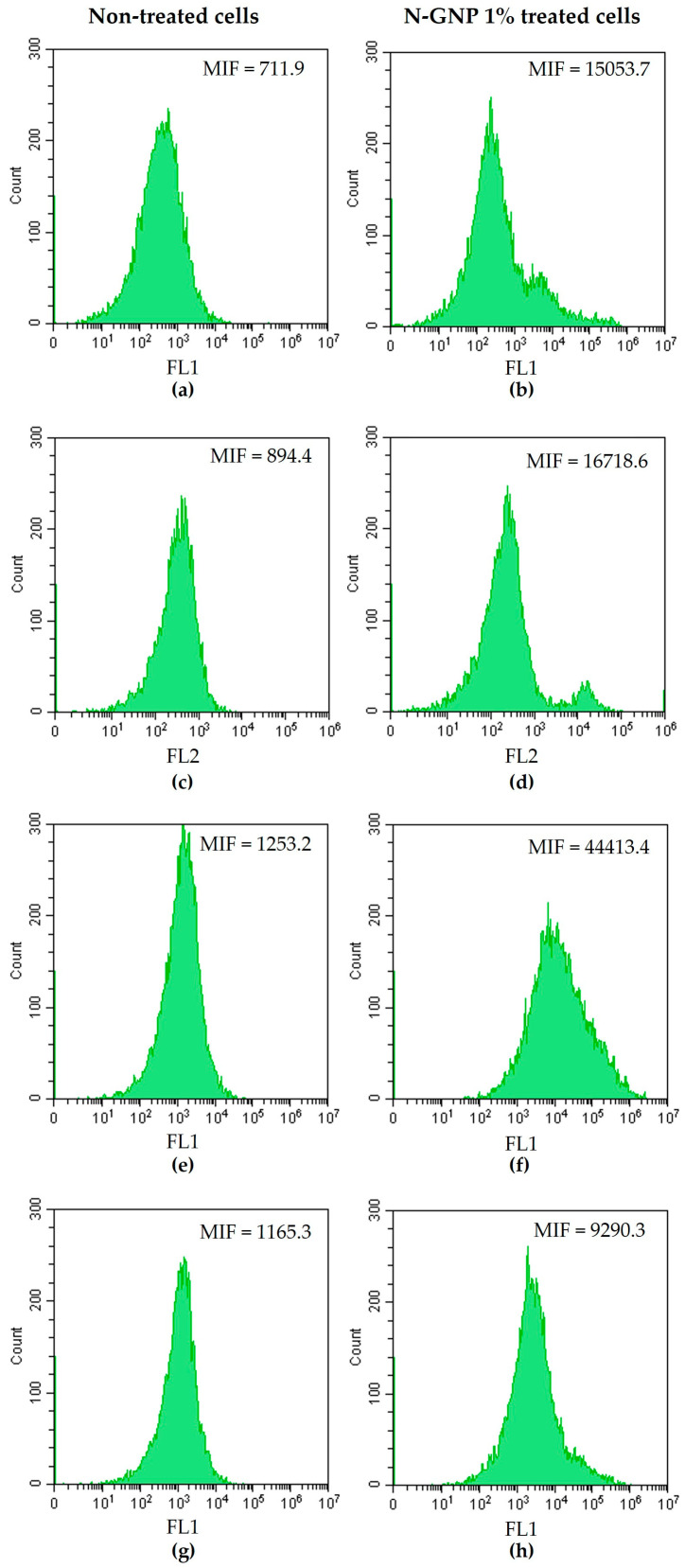
Representative flow cytometric histograms obtained for *S. aureus* non-treated and treated with 1% (*w*/*v*) N-GNP, stained with (**a**,**b**) DiBAC_4_(3), (**c**,**d**) PI, (**e**,**f**) 5-CFDA, and (**g**,**h**) DCFH-DA, respectively. Results were presented as the mean intensity of fluorescence (MIF).

**Figure 13:**
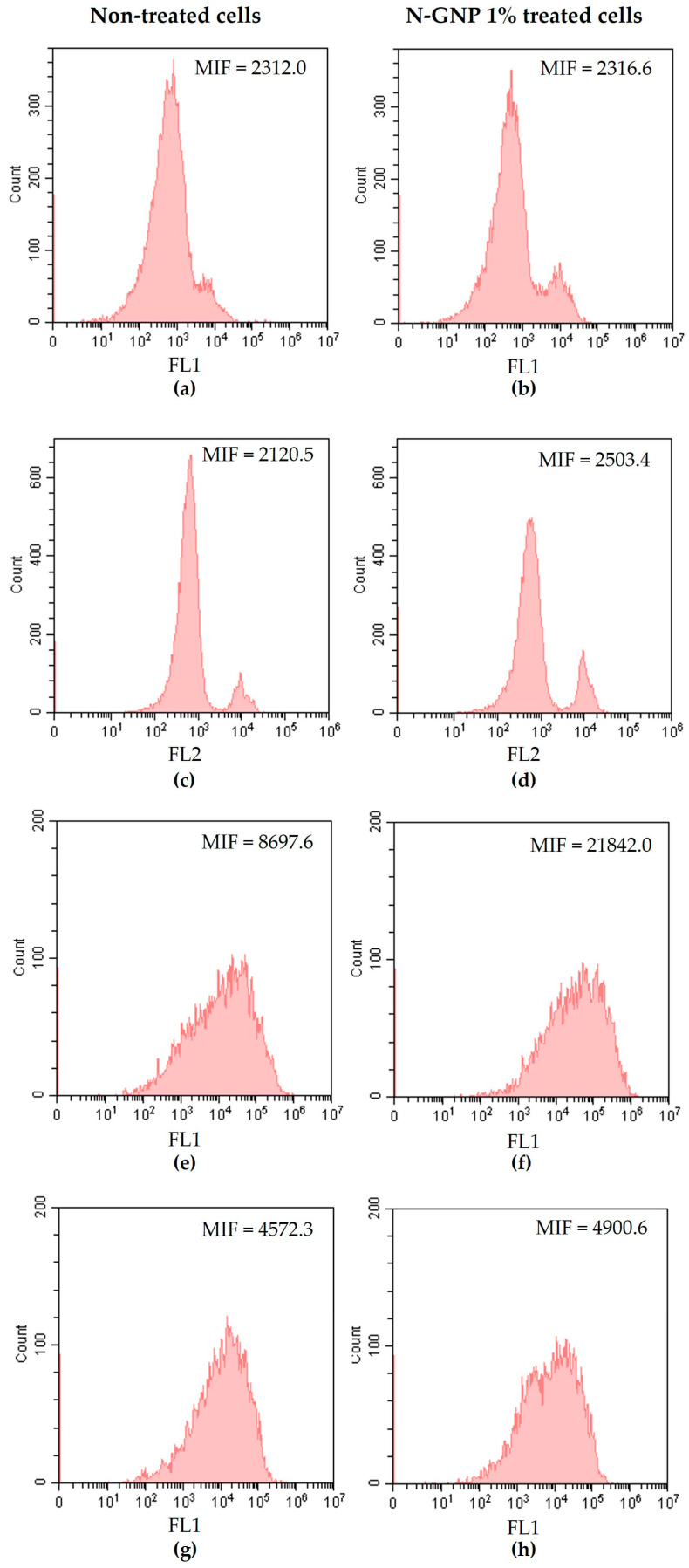
Representative flow cytometric histograms obtained for *P. aeruginosa* non-treated and treated with 1% (*w*/*v*) N-GNP, stained with (**a**,**b**) DiBAC_4_(3), (**c**,**d**) PI, (**e**,**f**) 5-CFDA, and (**g**,**h**) DCFH-DA, respectively. Results were presented as the mean intensity of fluorescence (MIF).

**Figure 14:**
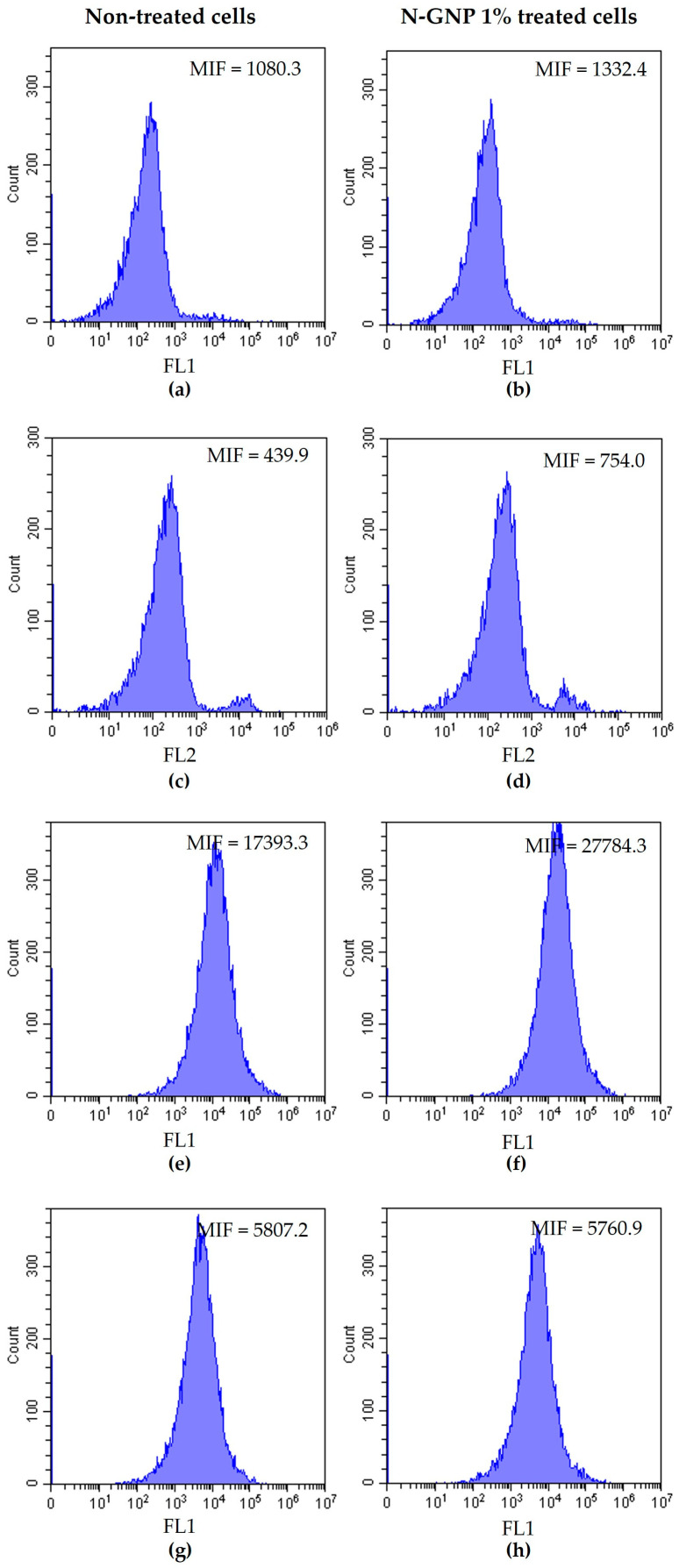
Representative flow cytometric histograms obtained for *K. pneumoniae* non-treated and treated with 1% (*w*/*v*) N-GNP, stained with (**a**,**b**) DiBAC_4_(3), (**c**,**d**) PI, (**e**,**f**) 5-CFDA, and (**g**,**h**) DCFH-DA, respectively. Results were presented as the mean intensity of fluorescence (MIF).

Numerous research studies have documented the promising antibacterial potential of graphene and its derivatives [18,24,27]. However, their mechanisms of action are poorly documented and require further investigation [61]. In order to characterize the antibacterial mechanisms of N-functionalized graphene, *S. aureus*, *P. aeruginosa*, and *K. pneumoniae* were treated with 1% (*w*/*v*) N-GNP for 24 h and stained with DiBAC_4_(3) (a membrane depolarization marker), PI (a membrane integrity marker), 5-CFDA (a metabolic activity marker), and DCFH-DA (a ROS production indicator), and analyzed by flow cytometry (Figure 12, Figure 13 and Figure 14). Non-treated bacteria stained with the aforementioned dyes were used as controls.

Flow cytometry data indicated that *S. aureus* treated with N-GNP exhibited changes in the cell membrane, as demonstrated by DiBAC_4_(3) and PI staining (Figure 12a–d). Cells stained with these two dyes displayed a higher mean intensity of fluorescence (MIF) than non-treated cells (approximately 20-fold higher). This finding is aligned with the proposed GNP mechanism of action, which hypothesizes that the sharp edges of GNP nanosheets physically damage bacterial membranes [35,62]. It was also observed that N-GNP increased *S. aureus* metabolic activity compared to non-treated cells, as demonstrated by the increase in MIF of cells stained with 5-CFDA (approximately 20-fold higher; Figure 12e,f). This suggests that bacteria reprogrammed their metabolism to counteract the stress imposed by graphene. Moreover, *S. aureus* treated with N-GNP and stained with DCFH-DA presented higher MIF than non-treated cells (8-fold higher, Figure 12g,h), indicating ROS production. Indeed, some studies have demonstrated that exposure to graphene-based materials increases ROS production in bacteria, which, in turn, contributes to cell membrane damage and, ultimately, bacterial death [63]. Altogether, these data indicate that GNP targets the *S. aureus* cell membrane and induces ROS production.

**Figure 15:**
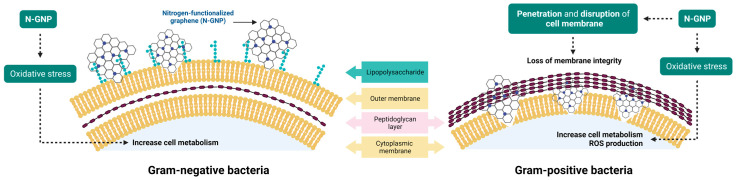
Schematic representation of the N-GNP antibacterial mechanisms against Gram-negative and Gram-positive bacteria. In Gram-negative bacteria, N-GNP increases cell metabolism as response to oxidative stress; whereas, in Gram-positive bacteria, this carbon material causes loss of membrane integrity, induces changes in cell metabolism and triggers ROS production.

Concerning Gram-negative bacteria (*P. aeruginosa* and *K. pneumoniae*), N-GNP did not cause substantial changes in cell membrane integrity, as demonstrated by DiBAC_4_(3) and PI staining (similar MIF values between treated and non-treated cells were found; Figure 13a–d and Figure 14a–d). Moreover, since there were no differences in MIF of N-GNP-treated and non-treated cells stained with DCFH-DA, it can be assumed that, under the tested conditions, this carbon material did not induce ROS production (Figure 13g,h and Figure 14g,h). However, when exposed to N-GNP, both *P. aeruginosa* and *K. pneumoniae* suffered changes in their metabolic activities, as revealed by 5-CFDA staining (2.5- and 1.5-fold higher MIF for *P. aeruginosa* and *K. pneumoniae* treated cells, respectively; Figure 13e,f and Figure 14e,f), suggesting that the bacteria are trying to adapt to the hostile environment imposed by N-GNP. A schematic representation of the N-GNP action mechanisms against Gram-positive and Gram-negative bacteria is presented in Figure 15.

Overall, these data corroborate the antimicrobial performance of the 1 wt. % N-GNP/PDMS surface against *S. aureus* to the detriment of *P. aeruginosa* and *K. pneumoniae*. A variety of experimental conditions can influence the antimicrobial activity of graphene materials, including the bacteria type (rod or spherical), since microorganisms have different morphological structures and abilities for growth under certain physiological conditions [27].

## 4. Conclusions

This study demonstrated that biofilm formation processes were influenced not solely by surface characteristics (e.g., roughness and hydrophobicity) but also by the tested bacteria’s properties. The 1 wt. % N-GNP/PDMS composite showed a high potential to be used as a coating for UCs since this surface significantly inhibited single- and multi-species biofilms formed by different uropathogenic organisms when compared to bare PDMS. The antibiofilm and antimicrobial activities of this graphene-based surface were particularly noticeable against the Gram-positive *S. aureus* strain. Hence, this composite could significantly contribute to diminishing the occurrence of CAUTIs and the therapeutic failure of UCs. However, while the potential of N-GNP/PDMS surfaces for biofilm prevention is promising, there are still challenges to overcome, such as scalability, cost-effectiveness, long-term stability, and biocompatibility, which must be addressed before real-life applications. Additionally, the coating must be further optimized (by introducing additional functionalization in the graphene or by the inclusion of other compounds) so that its efficacy against Gram-negative bacteria can be improved.

## Figures and Tables

**Table 1:** Elemental composition (wt. %) of graphene samples (p-GNP and N-GNP).

Sample	C	H	N	S	O
p-GNP	90.80 ± 0.80	0.44 ± 0.01	0.03 ± 0.00	0.05 ± 0.03	5.60 ± 0.07
N-GNP	88.60 ± 0.20	0.58 ± 0.03	4.17 ± 0.02	0.02 ± 0.00	3.80 ± 0.12

**Table 2:** Textural properties of p-GNP and N-GNP.

Property	Sample	
	p-GNP	N-GNP
S_BET_ (m^2^ g^−1^)	471	361
V_micro_ (cm^3^ g^−1^)	0.052	0.073
S_meso_ (m^2^ g^−1^)	353	196
V_P_ (p/p_0_ = 0.99) (cm^3^ g^−1^)	0.892	0.485

**Table 3:** Roughness parameters (*S_a_* and *S_q_*) of PDMS and 1 wt. % N-GNP/PDMS. Results are presented as mean ± SD.

Sample	Roughness (µm)	
	*S_a_*	*S_q_*
PDMS	47.5 ± 8.4	75.3 ± 6.9
1 wt. % N-GNP/PDMS	155.8 ± 33.2	236.1 ± 73.4

Nomenclature: *S_a_*—arithmetical mean height; *S_q_*—root mean square height.

**Table 4:** Contact angles and surface hydrophobicity of PDMS and 1 wt. % N-GNP/PDMS. Results are presented as mean ± SD. Significant differences between the contact angles on the tested surfaces were considered for *p* < 0.05 (*).

Sample	Contact Angle (°)			Hydrophobicity (mJ m^−2^)
	*θ_W_*	*θ_F_*	*θ_B_*	*ΔG*
PDMS	114.3 ± 1.4	111.8 ± 1.6	88.3 ± 3.8	−63.1
1 wt. % N-GNP/PDMS	122.1 ± 0.6 *	111.5 ± 1.7	64.7 ± 5.8 *	−81.1

Nomenclature: *θ_W_*, water contact angle; *θ_F_*, formamide contact angle; *θ_B_*, α-bromonaphthalene contact angle; *ΔG*, free energy of interaction between two entities of a given surface when immersed in water.

## Data Availability

The data presented in this study are available from the corresponding author upon request.

## Supplementary Materials

The following supporting information can be downloaded at: https://www.mdpi.com/article/10.3390/nano13182604/s1, Figure S1. Number of total cells of *S. aureus* biofilms formed on PDMS, 5 wt. % GNP/PDMS and 1, 3 and 5 wt. % N-GNP/PDMS surfaces. Results are presented as mean ± SD. Significant differences between GNP-based surfaces and the control (PDMS) were considered for *p*-values < 0.05 (*). Figure S2. Proportion of *P. aeruginosa* (in red), *S. aureus* (in green), and *K. pneumoniae* (in blue) cells in multi-species biofilms formed on PDMS (left) and 1 wt. % N-GNP/PDMS (right) surfaces. Figure S3. UV–vis spectra of AUM medium after contact with PDMS (green line) and 1 wt. % N-GNP/PDMS (blue line) surfaces.

## References

[1] McCleskey S.G., Shek L., Grein J., Gotanda H., Anderson L., Shekelle P.G., Keeler E., Morton S., Nuckols T.K. (2022). Economic evaluation of quality improvement interventions to prevent catheter-associated urinary tract infections in the hospital setting: A systematic review. BMJ Qual. Saf..

[2] Rhee C., Phelps M.E., Meyer B., Reed W.G. (2016). Viewing Prevention of Catheter-Associated Urinary Tract Infection as a System: Using Systems Engineering and Human Factors Engineering in a Quality Improvement Project in an Academic Medical Center. Jt. Comm. J. Qual. Patient Saf..

[3] Vallejo-Torres L., Pujol M., Shaw E., Wiegand I., Vigo J.M., Stoddart M., Grier S., Gibbs J., Vank C., Cuperus N. (2018). Cost of hospitalised patients due to complicated urinary tract infections: A retrospective observational study in countries with high prevalence of multidrug-resistant Gram-negative bacteria: The COMBACTE-MAGNET, RESCUING study. BMJ Open.

[4] Simmering J.E., Tang F., Cavanaugh J.E., Polgreen L.A., Polgreen P.M. (2017). The Increase in Hospitalizations for Urinary Tract Infections and the Associated Costs in the United States, 1998–2011. Open Forum Infect. Dis..

[5] Gomes M., Gomes L.C., Teixeira-Santos R., Mergulhão F.J., Carlsen P.N. (2020). PDMS in Urinary Tract Devices: Applications, Problems and Potential Solutions. Polydimethylsiloxane: Structure and Applications.

[6] Victor A.H.J., Apolónia S., Ribeiro J.G.R., Araújo F.F., Bragança, Portugal, Apolónia C.S., Cefet, Reis R.C.A.d., Janeiro R.D. (2019). Study of PDMS characterization and its applications in biomedicine: A review. J. Mech. Eng. Biomech..

[7] Zhang H., Chiao M. (2015). Anti-fouling Coatings of Poly(dimethylsiloxane) Devices for Biological and Biomedical Applications. J. Med. Biol. Eng..

[8] Feneley R.C.L., Hopley I.B., Wells P.N.T. (2015). Urinary catheters: History, current status, adverse events and research agenda. J. Med. Eng. Technol..

[9] Trautner B.W., Darouiche R.O. (2004). Role of biofilm in catheter-associated urinary tract infection. Am. J. Infect. Control.

[10] del Pozo J.L., Patel R. (2007). The Challenge of Treating Biofilm-associated Bacterial Infections. Clin. Pharmacol. Ther..

[11] Jacobsen S.M., Stickler D.J., Mobley H.L., Shirtliff M.E. (2008). Complicated catheter-associated urinary tract infections due to *Escherichia coli* and *Proteus mirabilis*. Clin. Microbiol. Rev..

[12] Weiner-Lastinger L.M., Abner S., Edwards J.R., Kallen A.J., Karlsson M., Magill S.S., Pollock D., See I., Soe M.M., Walters M.S. (2020). Antimicrobial-resistant pathogens associated with adult healthcare-associated infections: Summary of data reported to the National Healthcare Safety Network, 2015–2017. Infect. Control Hosp. Epidemiol..

[13] Sycz Z., Tichaczek-Goska D., Jezierska-Domaradzka A., Wojnicz D. (2021). Are Uropathogenic Bacteria Living in Multispecies Biofilm Susceptible to Active Plant Ingredient-Asiatic Acid?. Biomolecules.

[14] Musco S., Giammò A., Savoca F., Gemma L., Geretto P., Soligo M., Sacco E., Del Popolo G., Li Marzi V. (2022). How to Prevent Catheter-Associated Urinary Tract Infections: A Reappraisal of Vico’s Theory—Is History Repeating Itself?. J. Clin. Med..

[15] Ballo M.K., Rtimi S., Pulgarin C., Hopf N., Berthet A., Kiwi J., Moreillon P., Entenza J.M., Bizzini A. (2016). In Vitro and In Vivo Effectiveness of an Innovative Silver-Copper Nanoparticle Coating of Catheters To Prevent Methicillin-Resistant *Staphylococcus aureus* Infection. Antimicrob. Agents Chemother..

[16] Rtimi S., Sanjines R., Pulgarin C., Kiwi J. (2016). Quasi-Instantaneous Bacterial Inactivation on Cu–Ag Nanoparticulate 3D Catheters in the Dark and Under Light: Mechanism and Dynamics. ACS Appl. Mater. Interfaces.

[17] Teixeira-Santos R., Gomes M., Gomes L.C., Mergulhão F.J. (2021). Antimicrobial and anti-adhesive properties of carbon nanotube-based surfaces for medical applications: A systematic review. iScience.

[18] Kumar P., Huo P., Zhang R., Liu B. (2019). Antibacterial Properties of Graphene-Based Nanomaterials. Nanomaterials.

[19] Soldano C., Mahmood A., Dujardin E. (2010). Production, properties and potential of graphene. Carbon.

[20] Armano A., Agnello S. (2019). Two-Dimensional Carbon: A Review of Synthesis Methods, and Electronic, Optical, and Vibrational Properties of Single-Layer Graphene. C.

[21] Yang K., Feng L., Liu Z. (2015). The advancing uses of nano-graphene in drug delivery. Expert Opin. Drug Deliv..

[22] Geetha Bai R., Ninan N., Muthoosamy K., Manickam S. (2018). Graphene: A versatile platform for nanotheranostics and tissue engineering. Prog. Mater. Sci..

[23] Soliman M., Sadek A.A., Abdelhamid H.N., Hussein K. (2021). Graphene oxide-cellulose nanocomposite accelerates skin wound healing. Res. Vet. Sci..

[24] Akhavan O., Ghaderi E. (2010). Toxicity of Graphene and Graphene Oxide Nanowalls Against Bacteria. ACS Nano.

[25] Staneva A.D., Dimitrov D.K., Gospodinova D.N., Vladkova T.G. (2021). Antibiofouling Activity of Graphene Materials and Graphene-Based Antimicrobial Coatings. Microorganisms.

[26] Jia P.P., Sun T., Junaid M., Yang L., Ma Y.B., Cui Z.S., Wei D.P., Shi H.F., Pei D.S. (2019). Nanotoxicity of different sizes of graphene (G) and graphene oxide (GO) in vitro and in vivo. Environ. Pollut..

[27] Mohammed H., Kumar A., Bekyarova E., Al-Hadeethi Y., Zhang X., Chen M., Ansari M.S., Cochis A., Rimondini L. (2020). Antimicrobial Mechanisms and Effectiveness of Graphene and Graphene-Functionalized Biomaterials. A Scope Review. Front. Bioeng. Biotechnol..

[28] Olborska A., Janas-Naze A., Kaczmarek Ł., Warga T., Halin D.S.C. (2020). Antibacterial Effect of Graphene and Graphene Oxide as a Potential Material for Fiber Finishes. Autex Res. J..

[29] Liu S., Wei L., Hao L., Fang N., Chang M.W., Xu R., Yang Y., Chen Y. (2009). Sharper and Faster “Nano Darts” Kill More Bacteria: A Study of Antibacterial Activity of Individually Dispersed Pristine Single-Walled Carbon Nanotube. ACS Nano.

[30] Rodrigues D.F., Elimelech M. (2010). Toxic Effects of Single-Walled Carbon Nanotubes in the Development of *E. coli* Biofilm. Environ. Sci. Technol..

[31] Rocha R.P., Gonçalves A.G., Pastrana-Martínez L.M., Bordoni B.C., Soares O.S.G.P., Órfão J.J.M., Faria J.L., Figueiredo J.L., Silva A.M.T., Pereira M.F.R. (2015). Nitrogen-doped graphene-based materials for advanced oxidation processes. Catal. Today.

[32] Soares O.S.G.P., Rocha R.P., Gonçalves A.G., Figueiredo J.L., Órfão J.J.M., Pereira M.F.R. (2015). Easy method to prepare N-doped carbon nanotubes by ball milling. Carbon.

[33] Gomes M., Gomes L.C., Teixeira-Santos R., Pereira M.F.R., Soares O.S.G.P., Mergulhão F.J. (2021). Optimizing CNT Loading in Antimicrobial Composites for Urinary Tract Application. Appl. Sci..

[34] Restivo J., Orge C.A., Guedes Gorito dos Santos A.S., Soares O.S.G.P., Pereira M.F.R. (2020). Nanostructured Layers of Mechanically Processed Multiwalled Carbon Nanotubes for Catalytic Ozonation of Organic Pollutants. ACS Appl. Nano Mater..

[35] Oliveira I.M., Gomes M., Gomes L.C., Pereira M.F.R., Soares O.S.G.P., Mergulhão F.J. (2022). Performance of Graphene/Polydimethylsiloxane Surfaces against S. aureus and *P. aeruginosa* Single- and Dual-Species Biofilms. Nanomaterials.

[36] Belo S., Sousa-Cardoso F., Teixeira-Santos R., Gomes L.C., Vieira R., Sjollema J., Soares O.S.G.P., Mergulhão F.J. (2023). Production and Characterization of Graphene Oxide Surfaces against Uropathogens. Coatings.

[37] van Oss C.J. (2006). Interfacial Forces in Aqueous Media.

[38] Gedas A., Draszanowska A., Bakker H.d., Diez-Gonzalez F., Simões M., Olszewska M.A. (2023). Prevention of surface colonization and anti-biofilm effect of selected phytochemicals against *Listeria innocua* strain. Colloids Surf. B.

[39] Brooks T., Keevil C.W. (1997). A simple artificial urine for the growth of urinary pathogens. Lett. Appl. Microbiol..

[40] Heydorn A., Nielsen A.T., Hentzer M., Sternberg C., Givskov M., Ersbøll B.K., Molin S. (2000). Quantification of biofilm structures by the novel computer program COMSTAT. Microbiology.

[41] Noites R., Pina-Vaz C., Rocha R., Carvalho M.F., Gonçalves A., Pina-Vaz I. (2014). Synergistic Antimicrobial Action of Chlorhexidine and Ozone in Endodontic Treatment. Biomed Res. Int..

[42] Kramer B., Muranyi P. (2014). Effect of pulsed light on structural and physiological properties of *Listeria innocua* and *Escherichia coli*. J. Appl. Microbiol..

[43] Rosenkranz A.R., Schmaldienst S., Stuhlmeier K.M., Chen W., Knapp W., Zlabinger G.J. (1992). A microplate assay for the detection of oxidative products using 2′,7′-dichlorofluorescin-diacetate. J. Immunol. Methods.

[44] Sing K. (1985). Reporting physisorption data for gas/solid systems with special reference to the determination of surface area and porosity (Recommendations 1984). Pure Appl. Chem..

[45] Donohue M.D., Aranovich G.L. (1998). Classification of Gibbs adsorption isotherms. Adv. Colloid Interface Sci..

[46] León V., Quintana M., Herrero M.A., Fierro J.L.G., Hoz A.d.l., Prato M., Vázquez E. (2011). Few-layer graphenes from ball-milling of graphite with melamine. Chem. Comm..

[47] Zheng S., Bawazir M., Dhall A., Kim H.E., He L., Heo J., Hwang G. (2021). Implication of Surface Properties, Bacterial Motility, and Hydrodynamic Conditions on Bacterial Surface Sensing and Their Initial Adhesion. Front. Bioeng. Biotechnol..

[48] Krsmanovic M., Biswas D., Ali H., Kumar A., Ghosh R., Dickerson A.K. (2021). Hydrodynamics and surface properties influence biofilm proliferation. Adv. Colloid Interface Sci..

[49] Borges I., Henriques P.C., Gomes R.N., Pinto A.M., Pestana M., Magalhães F.D., Gonçalves I.C. (2020). Exposure of Smaller and Oxidized Graphene on Polyurethane Surface Improves its Antimicrobial Performance. Nanomaterials.

[50] Oh J.K., Yegin Y., Yang F., Zhang M., Li J., Huang S., Verkhoturov S., Schweikert E.A., Perez-Lewis K., Scholar E.A. (2018). The influence of surface chemistry on the kinetics and thermodynamics of bacterial adhesion. Sci. Rep..

[51] Harkes G., Feijen J., Dankert J. (1991). Adhesion of *Escherichia coli* on to a series of poly(methacrylates) differing in charge and hydrophobicity. Biomaterials.

[52] Cerca N., Pier G.B., Vilanova M., Oliveira R., Azeredo J. (2005). Quantitative analysis of adhesion and biofilm formation on hydrophilic and hydrophobic surfaces of clinical isolates of *Staphylococcus epidermidis*. Res. Microbiol..

[53] Hwang G., Kang S., El-Din M.G., Liu Y. (2012). Impact of conditioning films on the initial adhesion of *Burkholderia cepacia*. Colloids Surf. B.

[54] Ramstedt M., Ribeiro I.A.C., Bujdakova H., Mergulhão F.J.M., Jordao L., Thomsen P., Alm M., Burmølle M., Vladkova T., Can F. (2019). Evaluating Efficacy of Antimicrobial and Antifouling Materials for Urinary Tract Medical Devices: Challenges and Recommendations. Macromol. Biosci..

[55] Ghanim R., Mohammad M., Abdul Hussein A. (2018). Antibacterial Activity and Morphological Characterization of Synthesis Graphene Oxide Nanosheets by Simplified Hummer’s Method. Biosci. Biotechnol. Res. Asia.

[56] Di Giulio M., Zappacosta R., Di Lodovico S., Di Campli E., Siani G., Fontana A., Cellini L. (2018). Antimicrobial and Antibiofilm Efficacy of Graphene Oxide against Chronic Wound Microorganisms. Antimicrob. Agents Chemother..

[57] Al-Thani R.F., Patan N.K., Al-Maadeed M.A. (2014). Graphene oxide as antimicrobial against two gram-positive and two gram-negative bacteria in addition to one fungus. OnLine J. Biol. Sci..

[58] Zhang G., Meredith T.C., Kahne D. (2013). On the Essentiality of Lipopolysaccharide to Gram-Negative Bacteria. Curr. Opin. Microbiol..

[59] Sun J., Rutherford S.T., Silhavy T.J., Huang K.C. (2022). Physical properties of the bacterial outer membrane. Nat. Rev. Microbiol..

[60] Rojas E.R., Billings G., Odermatt P.D., Auer G.K., Zhu L., Miguel A., Chang F., Weibel D.B., Theriot J.A., Huang K.C. (2018). The outer membrane is an essential load-bearing element in Gram-negative bacteria. Nature.

[61] Qiu J., Liu L., Qian S., Qian W., Liu X. (2021). Why does nitrogen-doped graphene oxide lose the antibacterial activity?. J. Mater. Sci. Technol..

[62] Radhi A., Mohamad D., Abdul Rahman F.S., Abdullah A.M., Hasan H. (2021). Mechanism and factors influence of graphene-based nanomaterials antimicrobial activities and application in dentistry. J. Mater. Res. Technol..

[63] Akhavan O., Ghaderi E., Akhavan A. (2012). Size-dependent genotoxicity of graphene nanoplatelets in human stem cells. Biomaterials.

