# Supplementary material for: Exploring Nitrogen-Functionalized Graphene Composites for Urinary Catheter Applications
Source: Nanomaterials (Basel). 2023 Sep 21;13(18):2604. doi: 10.3390/nano13182604 (PMC10536687; doi:10.3390/nano13182604)
Supplement: Supplementary file 1 [file nanomaterials-13-02604-s001.zip › nanomaterials-2562119-supplementary.pdf]

Supplementary Material

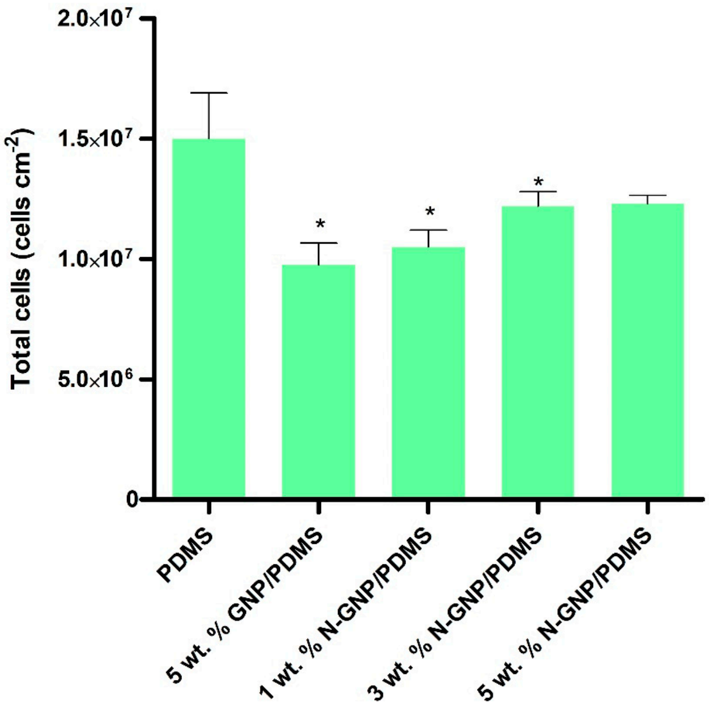

Figure S1. Number of total cells of *S. aureus* biofilms formed on PDMS, 5 wt. % GNP/PDMS and 1, 3 and 5 wt. % N-GNP/PDMS surfaces. Results are presented as mean ± SD. Significant differences between GNP-based surfaces and the control (PDMS) were considered for p-values < 0.05 (*).

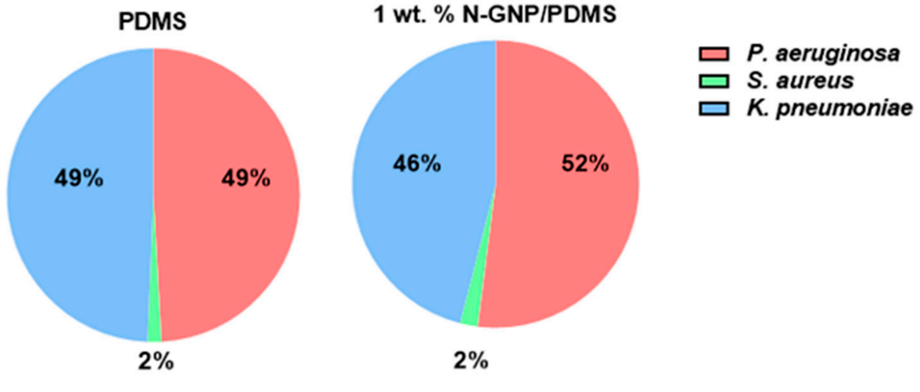

Figure S2. Proportion of *P. aeruginosa* (in red), *S. aureus* (in green), and *K. pneumoniae* (in blue) cells in multi-species biofilms formed on PDMS (left) and 1 wt. % N-GNP/PDMS (right) surfaces.

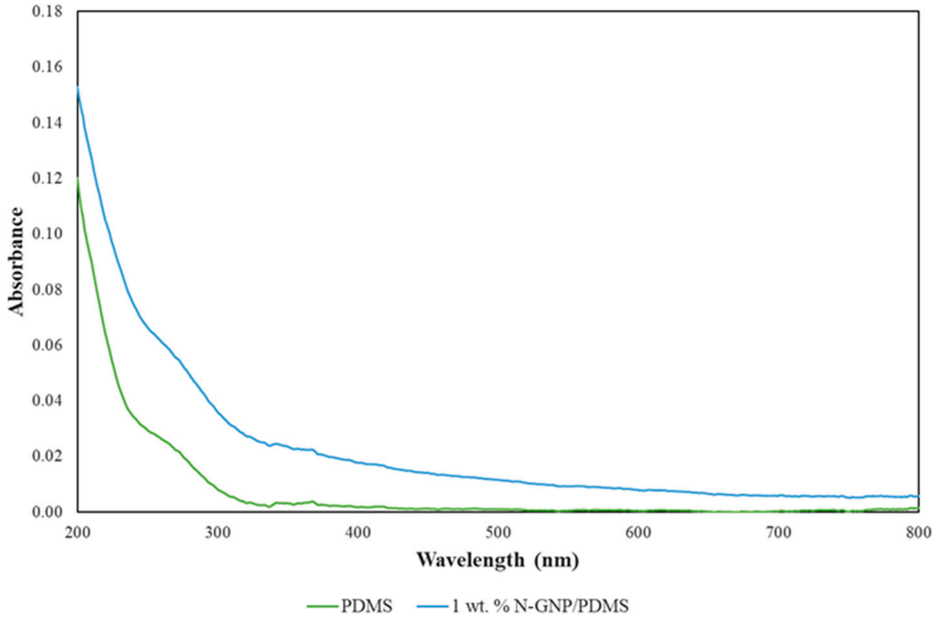

Figure S3. UV-Vis spectra of AUM medium after contact with PDMS (green line) and 1 wt. % N-GNP/PDMS (blue line) surfaces.
